# Transcranial direct current stimulation reverses neurophysiological and behavioural effects of focal inhibition of human pharyngeal motor cortex on swallowing

**DOI:** 10.1113/jphysiol.2013.263475

**Published:** 2013-12-13

**Authors:** Dipesh H Vasant, Satish Mistry, Emilia Michou, Samantha Jefferson, John C Rothwell, Shaheen Hamdy

**Affiliations:** 1 Gastrointestinal Centre, Institute of Inflammation and Repair, Manchester Academic Health Sciences Centre (MAHSC), University of Manchester, Salford Royal NHS Foundation TrustSalford, UK; 2 Sobell Department of Motor Neuroscience and Movement Disorders, Institute of Neurology, University College LondonLondon, UK

## Abstract

The human cortical swallowing system exhibits bilateral but functionally asymmetric representation in health and disease as evidenced by both focal cortical inhibition (pre-conditioning with 1 Hz repetitive transcranial magnetic stimulation; rTMS) and unilateral stroke, where disruption of the stronger (dominant) pharyngeal projection alters swallowing neurophysiology and behaviour. Moreover, excitatory neurostimulation protocols capable of reversing the disruptive effects of focal cortical inhibition have demonstrated therapeutic promise in post-stroke dysphagia when applied contralaterally. In healthy participants (*n* = 15, 8 males, mean age (±SEM) 35 ± 9 years), optimal parameters of transcranial direct current stimulation (tDCS) (anodal, 1.5 mA, 10 min) were applied contralaterally after 1 Hz rTMS pre-conditioning to the strongest pharyngeal projection. Swallowing neurophysiology was assessed in both hemispheres by intraluminal recordings of pharyngeal motor-evoked responses (PMEPs) to single-pulse TMS as a measure of cortical excitability. Swallowing behaviour was examined using a pressure-based reaction time protocol. Measurements were made before and for up to 60 min post intervention. Subjects were randomised to active or sham tDCS after 1 Hz rTMS on separate days and data were compared using repeated measures ANOVA. Active tDCS increased PMEPs bilaterally (*F*_1,14_ = 7.4, *P* = 0.017) reversing the inhibitory effects of 1 Hz rTMS in the pre-conditioned hemisphere (*F*_1,14_ = 10.1, *P* = 0.007). Active tDCS also enhanced swallowing behaviour, increasing the number of correctly timed challenge swallows compared to sham (*F*_1,14_ = 6.3, *P* = 0.025). Thus, tDCS to the contralateral pharyngeal motor cortex reverses the neurophysiological and behavioural effects of focal cortical inhibition on swallowing in healthy individuals and has therapeutic potential for dysphagia rehabilitation.

## Introduction

Deglutition is an essential gastrointestinal function with its motor control being bilaterally represented in the cerebral cortex (Hamdy *et al*. 1996, 1999a,*b*1999b). Evidence from studies of hemispheric stroke has highlighted the relevance of functional asymmetry in the swallowing motor network, with lesions affecting the ‘dominant’ (stronger pharyngeal representation) hemisphere leading to oropharyngeal dysphagia (Hamdy *et al*. 1997, 1998b; Khedr *et al*. 2008; Li *et al*. 2009; Teismann *et al*. 2011). Furthermore, re-organisation with increased pharyngeal representation in the non-dominant or weaker (unlesioned) hemisphere appears to be associated with recovery of swallowing function (Hamdy *et al*. 1998b; Li *et al*. 2009; Teismann *et al*. 2011). Indeed, the swallowing motor network has been shown to be adaptable to both peripheral and cortical stimuli and exhibits remarkable plastic change (Hamdy *et al*. 1998c; Gow *et al*. 2004; Mistry *et al*. 2012). Recently there has been much interest in both peripheral and cortical neurostimulation techniques to drive this neuroplastic process by targeting the contralesional cortex (Fraser *et al*. 2002; Khedr *et al*. 2009; Singh *et al*. 2009; Michou *et al*. 2012; Mistry *et al*. 2012; Park *et al*. 2013). Development of an inhibitory pre-conditioning protocol in the pharyngeal motor system has facilitated significant advances, allowing ‘preclinical’, first-in-man application of these neurostimulation techniques in a controlled environment to assess the efficacy of these interventions in a disrupted system before progressing to patient trials (Mistry *et al*. 2007; Jefferson *et al*. 2009a; Jayasekeran *et al*. 2010; Michou *et al*. 2012). Using this method, the investigator can focally inhibit the strongest pharyngeal corticobulbar projection, an intervention which has been shown to induce transient suppressive effects on swallowing neurophysiology and alter swallowing behaviour for up to 45 min, giving a window of opportunity to trial novel neurostimulation techniques (Mistry *et al*. 2007). Moreover, it has recently been shown using videofluoroscopy in healthy subjects that the application of this intervention can induce short-term effects on the physiological measurements of swallowing, reminiscent of deficits after stroke (Verin *et al*. 2012).

Transcranial direct current stimulation (tDCS) is a relatively new, non-invasive brain stimulation modality in which a small direct current is applied via scalp electrodes to polarise neurones in the underlying cortex (Nitsche & Paulus, 2000, 2001). Data from the stroke literature suggest that tDCS may have a role in expediting recovery of motor behaviour and procedural learning (Hummel *et al*. 2005; Schlaug *et al*. 2008; Stagg *et al*. 2012; Zimerman *et al*. 2012). tDCS has translational advantages compared to other cortical neurostimulation-based treatments that have been trialled in dysphagia rehabilitation, including its portability, ease of use, low costs and a less invasive intervention which in itself does not actually require pharyngeal intubation. These practical points make tDCS an attractive option for delivery at the bedside. Indeed, studies of anodal tDCS, when applied at either 1 mA for 20 min or 1.5 mA for 10 min (identified as the parameters which produced the largest effects at 60 min post intervention (Jefferson *et al*. 2009b), have been able to increase ipsilateral pharyngeal motor cortex excitability with effects comparable to other promising forms of neurostimulation such as pharyngeal electrical stimulation (Fraser *et al*. 2002) and rTMS (Jefferson *et al*. 2009a). Against this background, three small clinical studies using tDCS in post-stroke dysphagia have provided tantalising evidence for a useful role in dysphagic stroke but have been hampered by methodological inconsistencies including: hemisphere selected for stimulation, interventional parameters and swallowing behavioural outcome measures. A pilot study by Kumar *et al*. (2011) provided preliminaryevidence for immediate clinical effects of active contralateral tDCS on clinical severity of dysphagia scores, but used parameters previously untested in the pharyngeal system with limited measurable effects on swallowing behaviour. The other two clinical trials (Yang *et al*. 2012; Shigematsu *et al*. 2013) used evidence-based parameters of tDCS to stimulate the injured (lesioned) hemisphere. Only one of these studies included swallowing behavioural measurements and reported effects that took 3 months post intervention to build up (Yang *et al*. 2012). In summary, there is now a pressing need to perform studies based on robust methodological practice that will provide more information as to whether tDCS can be a useful therapeutic tool in the rehabilitation of dysphagia after stroke.

Given these clinical uncertainties, the aim of this study was to determine whether optimised parameters of contralateral tDCS are able to reverse the neurophysiological and behavioural effects of inhibitory pre-conditioning with 1 Hz rTMS applied to the strongest pharyngeal projection in healthy volunteers, as a prelude to applying this novel intervention in dysphagic stroke patients.

## Methods

### Subjects

Sample size calculation based on previous studies using the inhibitory pre-conditioning model within our department (Jefferson *et al*. 2009a; Jayasekeran *et al*. 2010; Michou *et al*. 2012) revealed that 12 subjects would be required to achieve a power of 80% and statistical significance of 5% (with standard deviation of 2.5). We therefore chose to recruit a minimum of 14 subjects to allow for drop-out.

Fifteen healthy volunteers (8 males, age range 21–61 years, mean (±SEM) 35 ± 9 years) completed the study. All subjects were in good health, our exclusion criteria being: history of epilepsy, cardiac pacemaker, previous brain surgery, previous swallowing problems, use of medication which acts on the central nervous system or implanted metal. This trial was ethically approved by Greater Manchester South Research Ethics Committee. Written informed consent was obtained from each volunteer prior to participation. All studies were conducted in accordance with the World Medical Association *Declaration of Helsinki*.

### Experimental procedures

#### Pharyngeal motor-evoked potentials (PMEPs)

Volunteers were required to pass a 3.2 mm diameter intraluminal catheter (Gaeltec Ltd, Dunvegan, Isle of Skye, UK) either transnasally or transorally, depending on their preference. The catheter houses a pair of bipolar platinum ring electrodes that were positioned in the pharynx to record electromyographic (EMG) traces. An earth was connected to a skin electrode sited over the upper portion of one of the sternocleidomastoid muscles in the neck.

#### Thenar motor-evoked potentials (TMEPs)

As a control, thenar EMG from the abductor pollicis brevis (APB) muscle contralateral to the hemisphere giving the largest PMEP was also recorded by TMS over the hand motor cortex. This was achieved using gel electrodes (H69P, Tyco Healthcare, Gosport, UK) placed 1 cm apart. An additional earth was connected to a skin electrode sited over a bony prominence on the wrist.

The catheter electrodes, thenar electrodes and the earths were all subsequently connected via a preamplifier (CED 1902; Cambridge Electronic Design, Cambridge, UK) with high and low pass filter settings of 200 Hz and 2 kHz, respectively, via connecting cables. Response signals were processed through a 50/60 Hz noise eliminator (‘HumBug’; Quest Scientific, North Vancouver, Canada) to remove any unwanted electrical interference collected through a laboratory interface (CED micro 1401) at a sampling rate of 5 kHz and recorded using Signal software (v4.0, CED) running on a personal computer.

#### Single-pulse transcranial magnetic stimulation (TMS)

Single TMS pulses were delivered using a figure-of-eight coil with an outer diameter of 7 cm, which produces a maximum output of 2.2 T (Magstim 200; The Magstim Company, Whitland, UK). The coil handle was held in antero-posterior direction at an angle of 45 deg tangential to the scalp as previously described (Hamdy *et al*. 1996).

#### Inhibitory pre-conditioning using repetitive transcranial magnetic stimulation (rTMS)

A Magstim super rapid stimulator (The Magstim Company) was used to deliver trains of stimuli through a figure-of-eight coil with a maximum output of 1.8 T. The Signal application software (CED) was programmed to deliver 1 Hz rTMS at 120% of pharyngeal resting motor threshold (rMT), limited to a maximum of 100% of stimulator output for 10 min (600 pulses in total) over the hemisphere which produced the largest amplitude PMEPs (strongest pharyngeal cortical projection; Mistry *et al*. 2007).

#### Transcranial direct current stimulation (tDCS)

tDCS was delivered using a custom-made device (Department of Medical Physics, Salford Royal NHS Foundation Trust). The polarity, intensity and duration settings of tDCS were based on the optimal excitatory regime defined by Jefferson *et al*. (1.5 mA of anodal tDCS for 10 min) given that these parameters produced the largest increase in cortical excitability at 60 min post intervention (Jefferson *et al*. 2009b). Interventions were delivered via two 25 cm^2^ rectangular surface electrodes (current density 0.06 mA cm^−2^). To ensure optimal contact with the scalp, both electrodes were placed in water-soaked pads (neuroConn GmbH, Ilmenau, Germany) and held in place by adjustable rubber straps. The anodal electrode was placed over the ‘unconditioned’ (see inhibitory pre-conditioning above) pharyngeal motor cortex and the other overlying the contralateral supraorbital ridge. For active tDCS, the current was slowly ramped up to 1.5 mA over 10 s, eliciting a transient tingling sensation. Impedance was monitored whilst stimulation continued for 10 min before being slowly turned off over 10 s. For sham tDCS, the current was turned off after 30 s, thus producing the same sensation as the active treatment but without significantly stimulating the cortex (Gandiga *et al*. 2006; Jefferson *et al*. 2009b).

#### Swallowing reaction task

The effects of tDCS and sham stimulation on swallowing behaviour were studied using an established experimental model as previously described by Mistry *et al*. (2007). For these experiments, a pharyngeal catheter incorporating a single solid-state pressure transducer (Gaeltec Ltd) was used. The catheter was connected to the interface, preamplifier and into the personal computer. Boluses of water, 3 ml in volume, were infused directly into the subject's oral cavity via a catheter connected to a hand-held syringe. A cutaneous electrical cue was generated using an electronic pulse generator (Digitimer, Welwyn Garden City, UK) connected to surface electrodes attached to the dorsum of the volunteer's hand. ‘Normal swallow’ reaction time was determined by asking participants to swallow at a normal pace after the cutaneous trigger. ‘Fast swallows’ required the volunteer to swallow as fast as possible after the cue. The latency from the electrical cue to the onset of the pharyngeal swallow, with consequent change in pharyngeal pressure signal, gave the reaction time measurement. From the recorded normal and fast swallowing reaction times, a challenge swallowing time window was calculated as described by Mistry *et al*. (2007). This challenge swallowing task is a visually cued, 150 ms time window on the laboratory desktop computer within which a swallow must be initiated to be successful.

#### Experiment 1. Effects of contralateral anodal tDCS on swallowing neurophysiology after pre-conditioning with 1 Hz rTMS to the stronger pharyngeal motor representation

**Figure 1:**
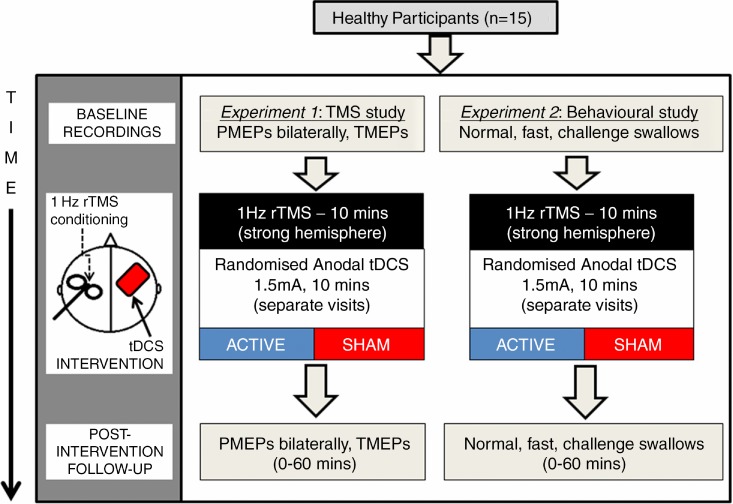
Abbreviations: PMEPs, pharyngeal motor-evoked potentials; TMEPs, thenar motor-evoked potentials.

Volunteers were randomised to receive active and sham tDCS interventions on two separate visits to the laboratory (Fig. 1), at least one week apart, using a randomisation programme (Stats Direct, v2.7.8, StatsDirect Ltd, Altrincham, UK).

During each session subjects were seated in a comfortable, reclining chair with the pharyngeal catheter *in situ*. The cranial vertex was marked on the scalp as a reference point. Single-pulse TMS was used at the start of each study to determine the strongest pharyngeal cortical projection and determine the optimal coil positions for recording PMEPs (the resting motor hot spots) over both hemispheres as well as the hand motor cortex in the stronger pharyngeal hemisphere. These sites were also marked on the scalp and the pharyngeal resting motor threshold (rMT) for each hemisphere was identified by using single pulses of stimulation to achieve evoked potentials of at least 20 μV on 50% of occasions. The pharyngeal motor cortex which produced the largest amplitude of PMEPs, at the lowest threshold, was defined as the ‘stronger’ pharyngeal hemisphere. Single-pulse TMS was then used to elicit TMEPs and determine thenar rMT on the side with the strongest pharyngeal representation.

Baseline measurements of cortical excitability at all three sites (stronger and weaker pharyngeal cortex and hand (thenar) motor cortex) were made by delivering 10 pulses of single-pulse TMS at rMT + 10% stimulator output and 10 pulses at rMT + 20% (60 stimuli in total). Following baseline measurements, volunteers all received inhibitory pre-conditioning to the strongest pharyngeal hemisphere, as described earlier. Either active or sham tDCS, dependent on the randomisation, was then delivered immediately after the completion of 1 Hz rTMS to the contralateral hemisphere, as detailed above, on two separate visits. Cortical excitability was then measured in the same way as with baseline, immediately and then repeated every 15 min for 1 h post intervention. Cortical excitability measurements post intervention were compared to baseline.

#### Experiment 2. Effects of contralateral anodal tDCS on swallowing behaviour following pre-conditioning with 1 Hz rTMS to the stronger pharyngeal motor representation

The swallowing behavioural studies were also conducted over two separate sessions (Fig. 1), with the same 15 subjects randomised to active and sham tDCS interventions at least one week apart. Volunteers were seated as in Expt 1 with the catheter housing the pressure sensors *in situ*. TMS was performed identically to Expt 1 in order to determine the PMEP hot spots and the strongest pharyngeal hemispheric projection. Baseline PMEP data were also recorded before proceeding to behavioural measurements. Volunteers performed 10 normal swallows followed by 10 fast swallows at baseline, with the volunteer swallowing 3 ml water each time. A challenging time window was then calculated via the software, with the volunteer required to perform the challenge task on 10 occasions. The volunteer's baseline challenged swallows score (number of correctly timed swallows out of 10) was subsequently recorded. Each subject then received inhibitory pre-conditioning (as in Expt 1) to the strongest pharyngeal projection. Immediately after 1 Hz rTMS conditioning, each volunteer received either active or sham tDCS to the unconditioned pharyngeal motor cortex as pre-determined by randomisation. Latencies for normal swallows and fast swallows, as well as the number of successful challenge swallows, were measured: immediately, 5, 10, 15, 30 and 60 min post tDCS intervention and compared to baseline.

### Data analysis

Experiment 1: the mean latencies and amplitudes of PMEPs and TMEPs were determined from each group of 10 EMG traces (for each site and intensity). In order to minimise variability, data were then normalised to baseline for each subject and expressed in the results as a percentage change from baseline. Experiment 2: the swallowing reaction time was defined as the interval between the onset of the stimulus to the hand and the time at which the pharyngeal pressure crossed a pre-determined threshold. The results for each set of normal and fast swallows were then averaged and normalised to baseline. The percentage change of correctly timed challenge swallows at each time point was also calculated by comparing the number of swallows where the pressure crossed the threshold within the set time window (out of 10) to baseline.

### Statistical methods

All data were analysed separately using a standard statistical software package (SPSS 20.0, SPSS Inc., Chicago, IL, USA). Initially, raw baseline MEP data from both experiments for the two interventions were compared separately using non-parametric (Wilcoxon signed rank) tests to exclude any bias resulting from the studies being conducted on separate days. Then, based on previous studies (Mistry *et al*. 2007, 2012; Jefferson *et al*. 2009a,*b*2009b; Jayasekeran *et al*. 2010; Michou *et al*. 2012) normalised (percentage change from baseline) MEP data from Expt 1 were compared using a general linear model repeated-measures analysis of variance (ANOVA), with factors of Treatment (active or sham tDCS), Hemisphere (conditioned or unconditioned) and Time (immediately, 15, 30 and 60 min post intervention). In Expt 2, normalised (percentage change from baseline) swallowing behavioural data were also compared using a general linear model repeated-measures ANOVA, with factors of Treatment (active or sham tDCS), and Time (immediately, 5, 10, 15, 30 and 60 min post intervention). In both experiments, when significant effects were present, these were followed up with *post hoc* analysis including adjustment of *P* values for multiple comparisons (Bonferroni correction) to explore the strength of the main effects. Non-sphericity was corrected using Greenhouse–Geisser where necessary. The above analyses were also performed for the MEP latency data using the raw values which displayed a normal distribution. *P* values < 0.05 were taken as a measure of statistical significance, and data are expressed as mean (± standard error of the mean (SEM)) unless stated otherwise.

## Results

In all 15 healthy volunteers; both TMS and rTMS were tolerated well with no adverse effects. Anodal tDCS (1.5 mA) for 10 min was also well tolerated and impedance was maintained below 8 kΩ in all subjects.

### Cortical hotspot mapping, resting motor thresholds and baseline TMS

**Figure 2:**
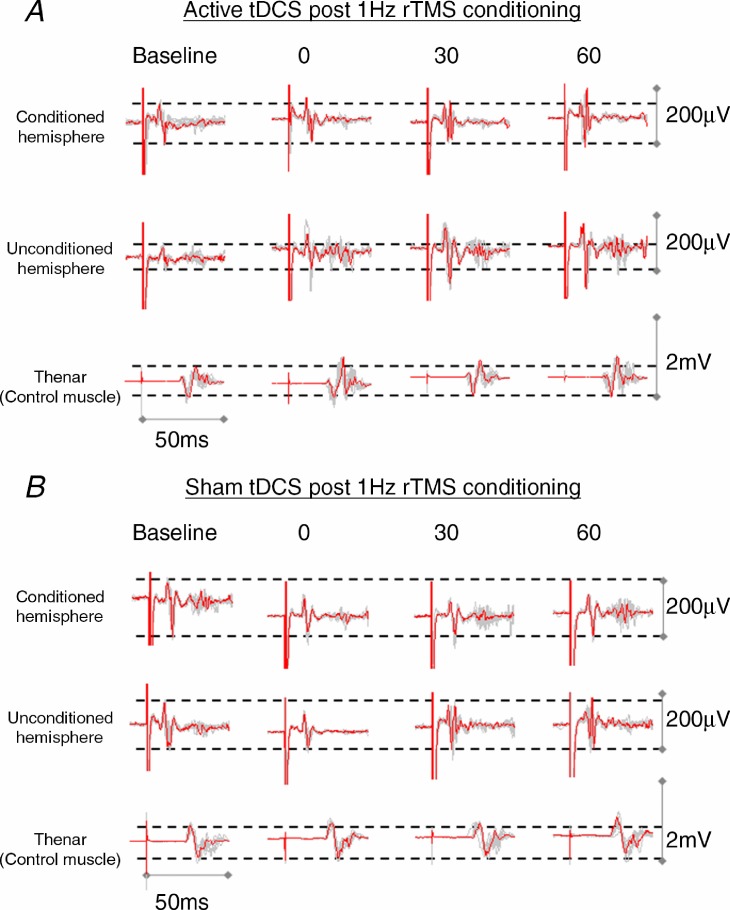
*A*, active tDCS post pre-conditioning with 1 Hz rTMS increased PMEP amplitudes bilaterally. *B*, sham tDCS post pre-conditioning with 1 Hz rTMS suppressed PMEPs on the conditioned hemisphere. TMEPs were not affected by either tDCS intervention. For visual purposes, responses from the intermediate time points 15 and 45 min post tDCS have been removed. Trace clusters for each recording site are composed of 10 overdrawn responses.

During single-pulse TMS mapping, 8/15 subjects were found to have stronger pharyngeal hemisphere representation on the left hemisphere whilst the other 7 subjects had stronger right hemispheric pharyngeal projections. The mean distances from the cranial vertex to the motor hot spots were: strong pharyngeal hemisphere, 3.2 ± 0.2 cm medio-lateral and 4.1 ± 0.2 cm antero-posterior; weaker pharyngeal projection, 3.1 ± 0.2 cm medio-lateral and 4.2 ± 0.3 cm antero-posterior; and thenar motor cortex representation, 3.5 ± 0.2 cm lateral and 4.0 ± 0.2 cm anterior. Mean rMT for strong pharyngeal hemisphere was 68 ± 3% stimulator output and 70 ± 3% stimulator output in the weaker pharyngeal hemisphere. Mean rMT for thenar motor cortex was 42 ± 2% stimulator output. The mean baseline PMEP amplitudes were 83 ± 5 μV for strong pharyngeal projection and 55 ± 4 μV over the weaker pharyngeal hemisphere. The mean baseline TMEP amplitudes were 772 ± 78 μV. There was no significant difference in baseline MEP data across the separate days (Wilcoxon signed rank test: strong pharyngeal projection, *Z* = −1.14, *P* = 0.26; weaker pharyngeal projection, *Z* = −0.51, *P* = 0.61; APB, *Z* = −0.22, *P* = 0.83). Figure 2 shows representative pharyngeal and thenar MEP data from one participant during the study.

#### Experiment 1. Effects of contralateral anodal tDCS on swallowing neurophysiology after pre-conditioning the strong pharyngeal motor cortex with 1 Hz rTMS

**Figure 3:**
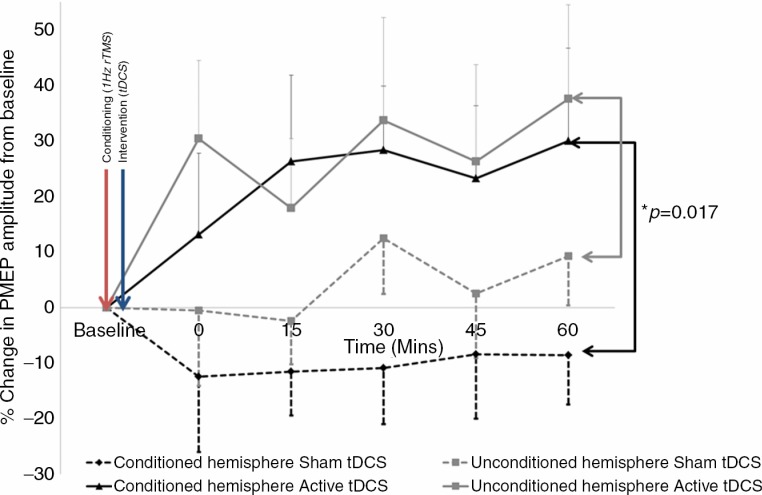
The dashed lines in this figure show the inhibitory changes induced by 1 Hz rTMS after sham tDCS. Active tDCS increases pharyngeal cortical excitability bilaterally (*P = 0.017).

rTMS of 1 Hz over the strong pharyngeal projection was tolerated well by all subjects with no adverse effects and was delivered at an average intensity of 96 ± 1% of rTMS output. Inhibitory pre-conditioning with 1 Hz rTMS, followed by contralateral sham anodal tDCS, suppressed cortical excitability in the conditioned hemisphere for the duration of the study (Fig. 3), with a decrease in PMEP amplitude of up to −13 ± 9%. However, inhibition in the unconditioned hemisphere was shorter (15 min), with a decrease in PMEP amplitude of only −2 ± 8%. By contrast, active tDCS post-inhibitory pre-conditioning increased PMEPs bilaterally (Fig. 3) by a maximum of 30 ± 17% in the conditioned hemisphere and 38 ± 17% in the unconditioned hemisphere.

A three-way repeated-measures ANOVA on normalised MEP data with factors of Treatment (active and sham tDCS), Site (conditioned pharyngeal hemisphere, unconditioned pharyngeal hemisphere and thenar cortex) and Time (immediately, 15, 30, 45 and 60 min post treatment) revealed a significant interaction of Treatment × Site × Time factors (*F*_1,14_ = 7.72, *P* = 0.015) and a significant effect of Treatment (*F*_1,14_ = 6.57, *P* = 0.023). A further three-way repeated measures ANOVA, this time with factors of Treatment (active and sham tDCS), Pharyngeal Hemispheres (conditioned and unconditioned) and Time (immediately, 15, 30, 45 and 60 min post treatment) confirmed significant effects of Treatment (mean difference in PMEPs of 30 ± 11%, 95% confidence interval of 6–53, *F*_1,14_ = 7.38, *P* = 0.017; adjusted for multiple comparisons: Bonferroni) on PMEPs but without differences in the pattern of excitability between Pharyngeal Hemispheres (*F*_1,14_ = 1.06, *P* = 0.32), implying that the significant effects of Treatment on PMEPs were bilateral. There were no significant effects of Time (*F*_4,56_ = 0.89, *P* = 0.48) and no other significant interactions were found.

When considering only the focally inhibited hemisphere, two-way repeated measures ANOVA with factors of Treatment and Time demonstrated a strong reversal effect by Treatment (mean difference in PMEPs of 35 ± 11%, 95% confidence level 11–58, *F*_1,14_ = 10.1, *P* = 0.007; adjusted for multiple comparisons: Bonferroni, Fig. 3).

The neurophysiological effects of contralateral tDCS did not, however, extend to the thenar motor cortex (two-way repeated measures ANOVA; no significant effects of Treatment (*F*_1,14_ = 0.83, *P* = 0.38), Time (*F*_2,29_ = 1.56, *P* = 0.23) or Treatment × Time (*F*_4,56_ = 0.79, *P* = 0.54)), therefore no further analyses were considered for the thenar data.

#### Experiment 1. The effects of tDCS on PMEP and TMEP latencies

**Figure 4:**
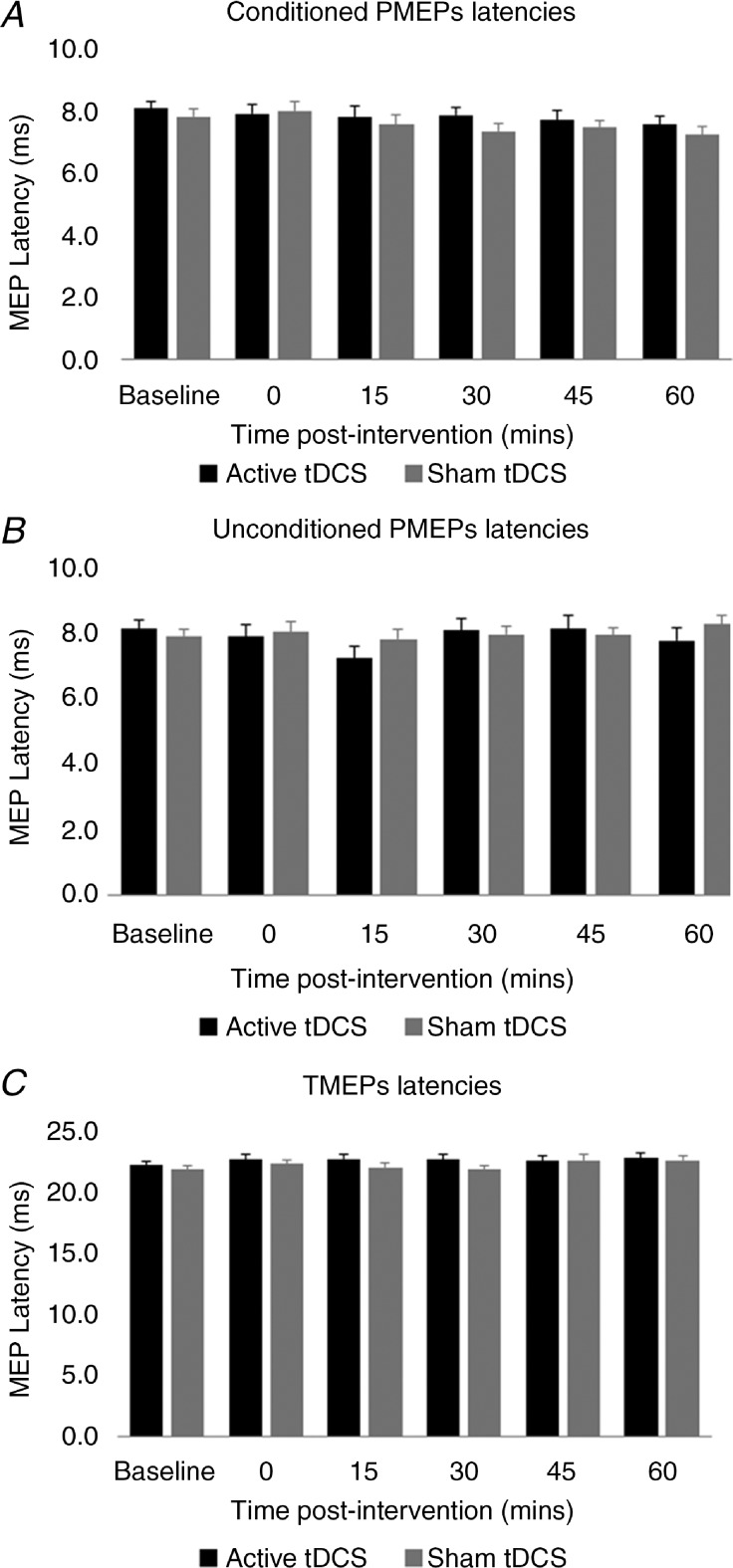
There were no significant effects of interventions on conditioned hemisphere PMEPs latencies (*A*), unconditioned hemisphere PMEPs latencies (*B*) or TMEPs latencies (*C*).

The mean response latencies at baseline and each time point for the PMEPs and TMEPs following tDCS are shown in Fig. 4. Wilcoxon signed rank tests comparing the raw baseline PMEP response latency values for each of the treatments (active tDCS and sham tDCS) for conditioned hemisphere (*Z* = −0.50, *P* = 0.62) and unconditioned hemisphere (*Z* = −0.54, *P* = 0.59) did not reveal any significant differences across the study days. There was also no significant difference in baseline TMEP latencies on the separate study days (*Z* = −0.94, *P* = 0.35).

Three-way repeated measures ANOVA did not reveal any significant effects of Treatment (*F*_1,14_ = 0.06, *P* = 0.81), Site (*F*_1,14_ = 3.2, *P* = 0.26) or Time (*F*_5,70_ = 1.3, *P* = 0.21) on PMEP latencies. Two-way repeated measures ANOVA revealed that there were also no significant effects of Treatment (*F*_1,14_ = 0.7, *P* = 0.20) or Time (*F*_5,70_ = 2.2, *P* = 0.07) on TMEP latencies.

#### Experiment 2. Effects of contralateral anodal tDCS on swallowing behaviour after pre-conditioning the strong pharyngeal motor cortex with 1 Hz rTMS

**Figure 5:**
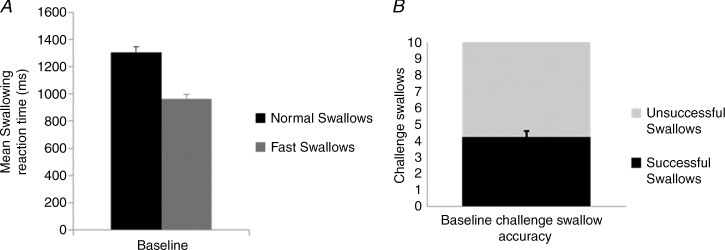
*A*, mean normal and fast swallowing reaction times (both visits). *B*, mean number of correctly timed challenge swallows (both visits).

Baseline TMS data collected prior to any interventions in Expt 2 confirmed that there was no difference in baseline PMEP amplitudes before receiving either active or sham tDCS on separate days (related-samples Wilcoxon signed rank test; strong pharyngeal projection, *Z* = −0.09, *P* = 0.93; weaker pharyngeal projection *Z* = −0.89, *P* = 0.37). There was no significant difference in baseline swallowing behavioural measures between the two separate sessions (related-samples Wilcoxon signed rank tests; Normal Swallows, *Z* = −1.13, *P* = 0.26; Fast Swallows, *Z* = −0.79, *P* = 0.43; and Challenge Swallows, *Z* = −1.79, *P* = 0.07). Grand mean (from both sessions) baseline reaction times for normal, fast swallows and challenge swallows data are displayed in Fig. 5.

**Figure 6:**
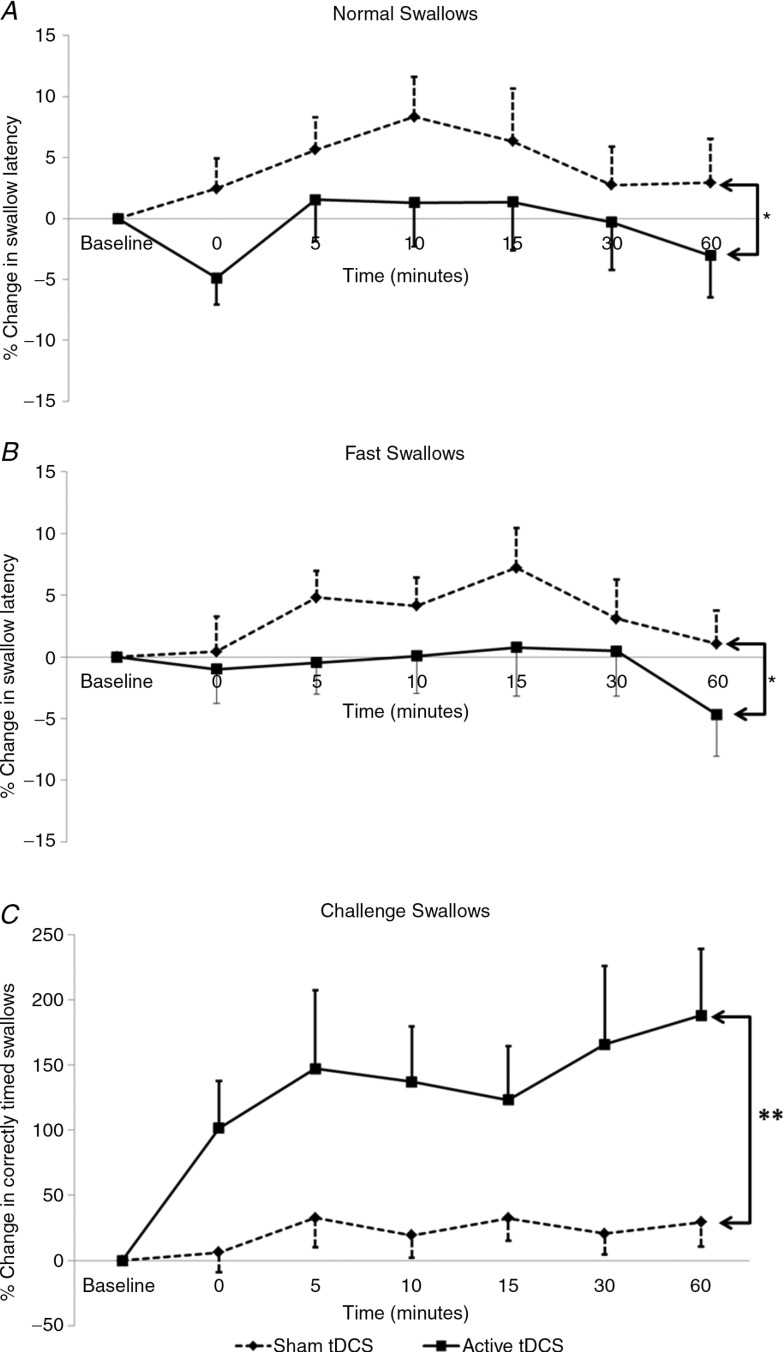
Graphs showing percentage change from baseline in normal swallow reaction times (*A*), fast swallow reaction times (*B*) and correctly timed challenge swallows (*C*) over 60 min post intervention. Active anodal tDCS significantly improved swallowing behaviour (*P = 0.03; **P = 0.025).

Normal and fast swallow latencies (expressed as percentage change from baseline) were analysed using a three-way repeated measures ANOVA with factors of Treatment (active or sham tDCS), Behaviour (normal or fast swallows) and Time (immediately, 5, 10, 15, 30 and 60 min post intervention). This revealed a significant effect of Treatment (mean change in reaction time: −5 ± 2%, 95% confidence interval −9 to −0.5, *F*_1,14_ = 5.62, *P* = 0.03; adjusted for multiple comparisons using Bonferroni correction, Fig. 6). However, there were no significant effects of Behaviour (mean change in reaction time: −0.2 ± 1.7%, 95% confidence interval −4 to +3.5, *F*_1,14_ = 2.2, *P* = 0.93) and no significant effects of Time when adjusting for multiple comparisons (Bonferroni) without any other significant interactions.

Active tDCS also improved the accuracy of the challenge swallow reaction time task with a mean improvement in correctly attempted swallows of +3.0 ± 0.6 out of 10 trials (+174% above baseline, Fig. 6*C*) at 60 min post intervention. In contrast, following sham tDCS there was virtually no improvement at the same time-point: +0.3 ± 0.6 out of 10 swallows (only +29% above baseline, Fig. 6*C*). Two-way repeated measures ANOVA on challenge swallow data (percentage change from baseline) revealed a significant effect of Treatment (mean difference: 119 ± 48%, 95% confidence interval 17–221, *F*_1,14_ = 6.3, *P* = 0.025; adjustment for multiple comparisons using Bonferroni correction) but no significant effects of Time (*F*_5,70_ = 1.31, *P* = 0.27) or Treatment × Time (*F*_5,70_ = 0.92, *P* = 0.47).

There was no correlation between findings in either experiment and relative inter-hemispheric asymmetry in pharyngeal projection (see online Supplemental data).

## Discussion

Our experiments examined the effects of tDCS on swallowing neurophysiology and behaviour after inhibitory pre-conditioning in an established model of brain suppression and swallowing disturbance in healthy participants. This model resembles the situation in unilateral stroke where patients with lesions affecting the strongest hemisphere often develop dysphagia. Interestingly, a sub-analysis of our data (see Supplementary Figure 1) similarly suggests that subjects with a greater degree of hemispheric asymmetry have greater neurophysiological and swallowing behavioural disruption after inhibitory pre-conditioning to the stronger pharyngeal projection. The observed effects of the active tDCS intervention to the unconditioned pharyngeal motor cortex were localised, with excitability of the hand motor cortex remaining unaltered.

### Bilateral reversal of focal cortical inhibition post anodal tDCS

Previous work from our group has shown that sham tDCS over the pharyngeal motor cortex does not alter cortical excitability in an unconditioned system (Jefferson *et al*. 2009b). Hence, as expected, sham tDCS following conditioning with 1 Hz rTMS to the strongest pharyngeal projection resulted in ipsilateral suppression of PMEPs that persisted throughout the 60 min of follow-up during Expt 1. By contrast, following active contralateral tDCS, there is a clear reversal in the direction of pharyngeal motor cortex excitability in the conditioned hemisphere. These sustained, bilateral excitatory effects peaked 60 min post stimulation. The increase in pharyngeal motor cortex excitability in the conditioned hemisphere implies that in an inhibited system, contralateral tDCS can more effectively produce transcallosal excitation. This is in contrast to the situation in an undisrupted system, where the same parameters of tDCS did not modulate pharyngeal cortical excitability in the opposite (unstimulated) hemisphere (Jefferson *et al*. 2009b). Previous studies suggest differing inter-hemispheric interactions in the bilaterally represented pharyngeal motor system, where both hemispheres appear to synergistically co-ordinate swallowing (albeit with functional asymmetry) (Hamdy *et al*. 1998a; Mistry *et al*. 2007), compared with the unilaterally innervated hand motor system, where transcallosal inhibition has been demonstrated (Ferbert *et al*. 1992). Following stroke affecting the hand motor areas, maladaptive increases in transcallosal inhibition from the unlesioned hemisphere have prompted investigators to attempt to counteract this with inhibitory tDCS protocols over the unlesioned hemisphere and applying anodal tDCS over the lesioned hemisphere in rehabilitation trials (Fregni *et al*. 2005; Boggio *et al*. 2007; Nowak *et al*. 2009). Given the lack of transcallosal inhibition in the pharyngeal system, the hand-stroke restorative model was not appropriate for the present study. We therefore targeted the unconditioned hemisphere as in previous inhibitory pre-conditioning studies of brain stimulation in the pharyngeal system (Jefferson *et al*. 2009a; Michou *et al*. 2012). Similar to our findings, the previous studies also demonstrated excitatory effects on the conditioned hemisphere, thereby increasing excitability of cortical projections to pharynx from both hemispheres. Our findings of increased excitability following contralateral tDCS in the hemisphere pre-conditioned with inhibitory rTMS are in accordance with previous work describing the phenomenon of homeostatic plasticity in the human motor cortex (Lang *et al*. 2004; Siebner *et al*. 2004; Cosentino *et al*. 2012). These studies demonstrated a strong shift in the direction of cortical excitability, when interventions ordinarily unable to enhance cortical excitability were preceded by inhibitory stimulation (Lang *et al*. 2004; Cosentino *et al*. 2012). Therefore, we propose that in a similar fashion, inhibitory (1 Hz) rTMS sensitised the conditioned cortical neurones before transcallosal spread of excitation via tDCS from the unconditioned hemisphere, provoking the reversal in direction of pharyngeal cortical excitability. During the early phase of recovery after stroke, similar homeostatic shifts in brain excitability have been described in lesioned areas with associated neurophysiological deficits (Murphy & Corbett, 2009; Carmichael, 2012). The same homeostatic shifts in brain excitability may therefore contribute to the measureable improvements in swallowing function seen in clinical trials of ipsilesional tDCS in post-stroke dysphagia (Yang *et al*. 2012; Shigematsu *et al*. 2013) whereby excitatory effects of lesioned hemisphere tDCS may be transmitted transcallosally to the unlesioned hemisphere, that being the hemisphere more closely implicated in swallowing recovery according to post-stroke dysphagia literature (Hamdy *et al*. 1998b; Li *et al*. 2009; Kumar *et al*. 2011; Teismann *et al*. 2011; Michou *et al*. 2012; Park *et al*. 2013).

One limitation of Expt 1 is that we did not measure pharyngeal cortical excitability between inhibitory pre-conditioning with 1 Hz rTMS and the contralateral tDCS intervention. Considering that a previous study has confirmed that sham tDCS does not alter pharyngeal cortical excitability (Jefferson *et al*. 2009b), our sham tDCS data in the present study indirectly demonstrate evidence for the inhibitory effects of 1 Hz rTMS. Inclusion of the additional time point between protocols may have provided further evidence for induction of focal cortical inhibition, homeostatic interactions and served as a secondary control for response variability between conditioning protocols.

Another limitation of Expt 1 is that we did not re-investigate the effects of anodal tDCS in an unconditioned system, where previously, anodal tDCS has already been demonstrated to increase pharyngeal motor excitability in the stimulated hemisphere only (Jefferson *et al*. 2009b). By contrast, in our study, in a pre-conditioned system after 1 Hz rTMS, we demonstrated bilateral effects of contralateral tDCS on pharyngeal excitability. These findings imply transcallosal spread of excitation to the opposite hemisphere. As discussed, the most plausible explanation for these findings is homeostatic plasticity after inhibitory pre-conditioning. Repeating the unconditioned experiments (i.e. after sham 1 Hz stimulation and anodal tDCS) in the present study would have strengthened our conclusions by enabling more direct (within-subject) comparisons of facilitation patterns between pharyngeal projections following anodal tDCS in pre-conditioned and unconditioned systems.

### Effects of reversing focal cortical inhibition by anodal tDCS on swallowing behaviour

In Expt 2, active tDCS following inhibitory pre-conditioning reduced both normal and fast swallowing reaction times. The physiological significance of this small but statistically supported reduction in normal and fast swallow latencies is unclear. Given that the increased velocity of these reaction time tasks was accompanied by a more accurate performance in the more complex challenged swallows, there is a suggestion that either the overall speeding up effect itself is beneficial or perhaps the by-product of a more co-ordinated and efficacious swallow post active tDCS. Using our pharyngeal pressure-based measures of normal and fast swallowing reaction times it is not possible to determine precisely which component of the swallow was influenced by reversal of focal cortical inhibition by tDCS. A videofluoroscopic study post 1 Hz rTMS to the stronger oropharyngeal projection has previously shown that focal cortical inhibition has differential effects on oral transit time (speeded up) and swallowing response times (delayed), without alteration in pharyngeal transit time or laryngeal closure duration (Verin *et al*. 2012). Our timings of normal and fast swallows would only capture the oral transit and the transitional phase between the oral and pharyngeal swallow (swallowing response time). Our findings therefore suggest that reversal of focal cortical inhibition may have improved the control and efficacy of the oral phase or reduced the 1 Hz rTMS-induced delay between the oral and pharyngeal phases of swallowing. Behavioural data from an unconditioned system in healthy subjects (Suntrup *et al*. 2013) which found no effects of anodal tDCS on normal and fast swallows imply that our findings result from reversal of the inhibitory pre-conditioning. Similar to the limitations of the neurophysiological experiment, we did not re-examine the behavioural effects of anodal tDCS in an unconditioned system which would have helped confirm this in the same group of subjects. A future study incorporating videofluoroscopic swallowing studies both in conditioned and unconditioned systems would help elucidate precisely which specific components of deglutative behaviour and timings are affected by anodal tDCS.

Compared to the normal and fast swallowing reaction time tasks, the challenge swallows are a more complex motor task, requiring processing of sensory cues and co-ordination of pharyngeal muscular activity within the 150 ms time window. After active tDCS, our data clearly show positive effects on swallowing behaviour, with a significant improvement in the number of correctly timed challenge swallows compared to sham. Given the progressive improvement in swallowing accuracy over time with maximum effects at the end of follow-up, these data demonstrate consolidation of motor learning and skill acquisition with repetition over time. Our behavioural data in a disrupted/conditioned system are in accordance with recently published findings in an unconditioned system in healthy subjects, where active tDCS combined with an oral motor and sensory task improved challenged swallowing behaviour (Suntrup *et al*. 2013). In the present study, each subject's first exposure to the swallowing reaction time protocol (and indeed their only training) was the 10 trials of each task during baseline recordings. tDCS stimulation was then administered ‘offline’, i.e. without any swallowing training taking place during stimulation. Our observations are in keeping with the studies of hand motor tasks where both ‘online’ (Nitsche *et al*. 2003; Galea & Celnik, 2009; Reis *et al*. 2009; Kang & Paik, 2011) and ‘offline’ (Tecchio *et al*. 2010) anodal tDCS has been shown to enhance performance.

### Mechanism of action of tDCS

Mechanistic studies to date suggest that anodal tDCS-induced increases in excitability result from depolarisation of cortical neurones and subsequent changes in resting membrane potential (Gomez Palacio Schjetnan *et al*. 2013). Pharmacological studies have demonstrated that anodal tDCS-induced increases in MEPs are dependent on synaptic sodium and calcium conductance and suggest that the long-lasting after-effects on cortical excitability may be dependent on glutamatergic NMDA receptors (Liebetanz *et al*. 2002; Nitsche *et al*. 2003*a*). Additionally, one magnetic spectroscopic study suggests decreases in GABAergic inhibition following anodal tDCS (Stagg *et al*. 2009). Functional magnetic resonance imaging studies have shown that tDCS to the motor cortex induced changes in neuroplasticity that can alter functional connectivity within the human brain (Polania *et al*. 2011; Kim *et al*. 2012). Therefore when tDCS is specifically applied to the pharyngeal motor cortex, our findings lead us to hypothesise that the increased cortical excitability in the pharyngeal motor areas may facilitate strengthening of task-related synapses in the swallowing motor network by enhancing functional coupling between the various cortical regions involved in swallowing. Recently published magnetoencephalography (MEG) data in healthy subjects provides further evidence for this, showing increased activity of several cortical regions involved in the planning, initiation and execution of swallowing following tDCS to pharyngeal motor cortex (Suntrup *et al*. 2013). The authors paired swallowing training and sucking flavoured lollipop interventions with tDCS in an undisrupted system and reported bilateral increase in swallow-related brain activation on MEG after tDCS (Suntrup *et al*. 2013). This is contrary to tDCS without swallowing training, which only increases ipsilateral cortical excitability as measured by TMS in an undisrupted system (Jefferson *et al*. 2009b) and suggests that there may be added benefits of synergistic swallowing training with tDCS. Future TMS studies in both undisrupted and disrupted systems examining the neurophysiological effects of swallowing training alone, compared to tDCS alone and tDCS with training, would therefore be of value to test this hypothesis and further optimise tDCS interventions.

Recent evidence from animal literature suggests that cortical tDCS can have a facilitatory effect in subcortical structures (Bolzoni *et al*. 2013a,*b*2013b). With respect to the level of facilitation in the swallowing motor system to tDCS, there is some evidence from our data (and previous studies, e.g. Jefferson *et al*. 2009b) that anodal stimulation effects are predominantly due to intracortical neuronal excitation rather than at the brainstem level. Firstly, if the effects of active tDCS on PMEPs were due to increased excitability of bulbar motoneurones, then we would have expected a shortening of cortico-pharyngeal latency reflecting the excited motoneurones being nearer to threshold. However, in the present study there were no differences in cortico-pharyngeal latency following active and sham tDCS. Secondly, a previous study in an uninhibited system has shown that tDCS only increased MEPs ipsilaterally in the pharyngeal motor system (Jefferson *et al*. 2009b) and if these changes were at the motoneurone level we would expect the MEP effects to be the same bilaterally as bulbar neurones receive input from both hemispheres. These observations make it unlikely that tDCS directly affected the brainstem, but in the absence of intra-brainstem recording, this assertion remains uncertain.

In conclusion, we have demonstrated that optimised parameters of anodal tDCS (without swallowing training) over the unconditioned hemisphere can restore swallowing physiology and behaviour to a disrupted system after inhibitory pre-conditioning to pharyngeal motor cortex. These results are of physiological and clinical relevance and suggest that 10 min of anodal tDCS at 1.5 mA has therapeutic potential as an adjunctive treatment for post-hemispheric stroke dysphagia when applied contralesionally and supports its application in future randomised clinical trials using these parameters. We have demonstrated that tDCS is a safe modality and is well tolerated at these parameters in healthy participants. Indications from small clinical trials of anodal tDCS, despite varying stimulation sites (Yang *et al*. 2012; Shigematsu *et al*. 2013) and parameters (Kumar *et al*. 2011) also suggest that this intervention would be safe in post-stroke dysphagia patients. A future clinical trial applying 1.5 mA anodal tDCS for 10 min contralesionally in post-stroke dysphagia patients will be required to confirm this. Further unanswered questions requiring investigation include the optimal number of treatment sessions required to facilitate recovery of swallowing function in a post-stroke dysphagia patient population. Therefore a randomised controlled dose–response study incorporating videofluoroscopic swallowing studies in a methodologically robust protocol would be an important step in determining the optimal dosage of contralesional tDCS. Data in the present study suggest that tDCS may enhance motor memory acquisition resulting in improved swallowing behaviour, therefore future trials in healthy participants and patients may explore the role of standardised swallowing training during active tDCS intervention compared to active tDCS without training and sham tDCS with training.

## Key points

- Cortical control of swallowing exhibits functional asymmetry with brain lesions involving the strongest projection being implicated in the pathophysiology of dysphagia after unilateral stroke.
- Swallowing recovery is associated with neuroplastic adaptation in the unlesioned hemisphere, a process which can be facilitated by excitatory neurostimulation techniques including transcranial direct current stimulation (tDCS).
- Unilateral suppression of the strongest pharyngeal motor projection using 1 Hz repetitive transcranial magnetic stimulation (rTMS) can disrupt swallowing neurophysiology and behaviour making it a useful model for trialling novel neurostimulation interventions in healthy subjects.
- In this healthy participant study we examined the effects of tDCS after unilateral pre-conditioning with 1 Hz rTMS to determine its ability to restore swallowing neurophysiology and behaviour.
- We show that application of optimised parameters of tDCS (anodal stimulation, 1.5 mA, 10 min) over the unconditioned hemisphere reverses the brain and behavioural consequences of inhibitory pre-conditioning, supporting the use of tDCS in clinical trials.

## Abbreviations

APB: abductor pollicis brevis
EMG: electromyography
MEG: magnetoencephalography
MEP: motor-evoked potential
PMEP: pharyngeal motor-evoked potential
rMT: resting motor threshold
rTMS: repetitive transcranial magnetic stimulation
tDCS: transcranial direct current stimulation
TMEP: thenar motor-evoked potential
TMS: transcranial magnetic stimulation

Translational perspective Using evidence-based parameters of transcranial direct current stimulation (tDCS) in a controlled environment and an established pre-clinical model of stroke we demonstrate therapeutically desirable increases in pharyngeal cortical excitability bilaterally, reversal of inhibitory pre-conditioning, and resultant improvement in swallowing behaviour in healthy subjects. Moreover this technique has been shown to be safe in healthy participants, non-invasive, portable, cost-effective and therefore has translational advantages over other forms of cortical neurostimulation. Additional research is required to develop this intervention further, investigating the mechanisms of neuroplasticity after tDCS to the pharyngeal motor cortex and obtaining dose–response data which are currently lacking. Our current data validate its application contralesionally; however, future translational studies in the dysphagic hemispheric stroke (and other neurological) patient populations would need to determine the optimal dosage whilst confirming its safety and efficacy.
