# Supplementary material for: Transcranial direct current stimulation reverses neurophysiological and behavioural effects of focal inhibition of human pharyngeal motor cortex on swallowing
Source: J Physiol. 2013 Dec 13;592(Pt 4):695–709. doi: 10.1113/jphysiol.2013.263475 (PMC3934709; doi:10.1113/jphysiol.2013.263475)
Supplement: Supplementary file 1 [file tjp0592-0695-sd1.docx]

**Supplementary Methods**

**Calculation of relative inter-hemispheric asymmetry between pharyngeal projections and correlation with neurophysiological and behavioural outcomes**

In order to quantify relative right-left asymmetry in pharyngeal projection we calculated a laterality index (LI) for each subject (n=15) based on mean baseline PMEPs amplitudes using the formula: (Right mean PMEPs – Left mean PMEPs)/ (Right mean PMEPs + Left mean PMEPs). The overall direction of laterality (plus = right, minus = left) was ignored for the purpose of this analysis.

For both *Experiment 1* data (conditioned and unconditioned PMEPs) and *Experiment 2* data (challenge swallows) we calculated mean post-tDCS percentage change using data from each post-intervention time point. Using Pearson bivariate correlation coefficient tested for correlation between LI scores and percentage changes post-tDCS in; conditioned hemisphere PMEPs after active tDCS and sham tDCS; unconditioned hemisphere PMEPs after active and sham tDCS and challenge swallows after active and sham tDCS.

**Subdivision of subjects into Laterality groups**

Based on the median LI score we divided our subjects into a lateralised and an unlateralised group. Using mean percentage change post-tDCS PMEPs data we then calculated a repeated measures ANOVA with factors of Treatment (active or sham tDCS), Pharyngeal Hemisphere (conditioned or unconditioned) with Laterality (Lateralised or Unlateralised) as a between-subjects factor. Similarly, for *Experiment 2* data we calculated repeated measures ANOVA on challenge swallows data with factors of Treatment and Laterality as a between-subjects factor.

**Supplementary Data**

**Relative asymmetry between pharyngeal projections and lack of correlation with neurophysiological and behavioural outcome measures**

The mean LI score was 0.22 ± 0.03 (range of 0.07 to 0.51) and a median of 0.2.

There was no significant correlation between LI scores and mean post tDCS percentage change in PMEPs (n=15) in the conditioned hemisphere after active (Pearson correlation -0.3, *p*=0.2) or sham tDCS (Pearson correlation -0.4, *p*=0.16). There were also no significant correlations with LI scores and unconditioned hemisphere PMEPs after active (Pearson correlation 0.3, *p*=0.9) or sham tDCS (Pearson correlation -0.3, *p*=0.4)

Similarly, there were no significant correlations between LI scores and mean post tDCS improvement in challenge swallows (Active tDCS Pearson correlation: 0.1, p=0.7, Sham tDCS Pearson Correlation: -0.4, *p*=0.1).

**Comparison of outcome measures between laterality groups**

Based on median LI score, we subdivided our subjects into those with LI>0.2 (lateralised n=7) and LI≤0.2 (n=8). Repeated measures ANOVA on mean post tDCS PMEP data with laterality as a between-subjects factor once again confirmed significant effects of Treatment (F_1, 13_=6.8, *p*=0.02), but without significant effects of Pharyngeal Hemisphere (F_1, 13_=0.99, *p*=0.33), or any Treatment x Laterality (F_1, 13_=0.07, *p*=0.80), Hemisphere x Laterality (F_1, 13_=0.004, *p*=0.95), or Treatment x Hemisphere x Laterality interactions ((F_1, 13_=0.38, *p*=0.55). On challenge swallows data, again we found a significant effect of Treatment (F_1, 13_=6.5, *p*=0.02), but no significant Treatment x Laterality interaction (F_1, 13_=0.64, *p*=0.44).

**Comparing the effectiveness of 1 Hz rTMS pre-conditioning in disrupting swallowing neurophysiology and behaviour between laterality groups**

Compared to unlateralised subjects, sham tDCS following 1 Hz to the strong pharyngeal projection produced greater neurophysiological deficit to the pre-conditioned hemisphere (Mann-Whitney *U* test; K=516, median difference (PMEP % change from baseline)= -22.9, *U*=386, *p*=0.0009, Supplementary Figure 1A) and greater disruption to challenged swallowing behaviour (Mann-Whitney-*U* test; K=766, median difference (% change challenge swallows from baseline)= +25, *U*=749.5, p=0.04 Supplementary Figure 1B) in lateralised subjects.

**Figure legends**

**Supplementary Figure 1: The effects of 1Hz pre-conditioning following sham tDCS were stronger in subjects with a Laterality Index >0.2 on;**

A) Conditioned hemisphere PMEPs (*p*<0.01)

B) Challenged swallow behaviour (*p*=0.04)
