# Supplementary figures and images for: Transcranial direct current stimulation reverses neurophysiological and behavioural effects of focal inhibition of human pharyngeal motor cortex on swallowing
Source: J Physiol. 2013 Dec 13;592(Pt 4):695–709. doi: 10.1113/jphysiol.2013.263475 (PMC3934709; doi:10.1113/jphysiol.2013.263475)

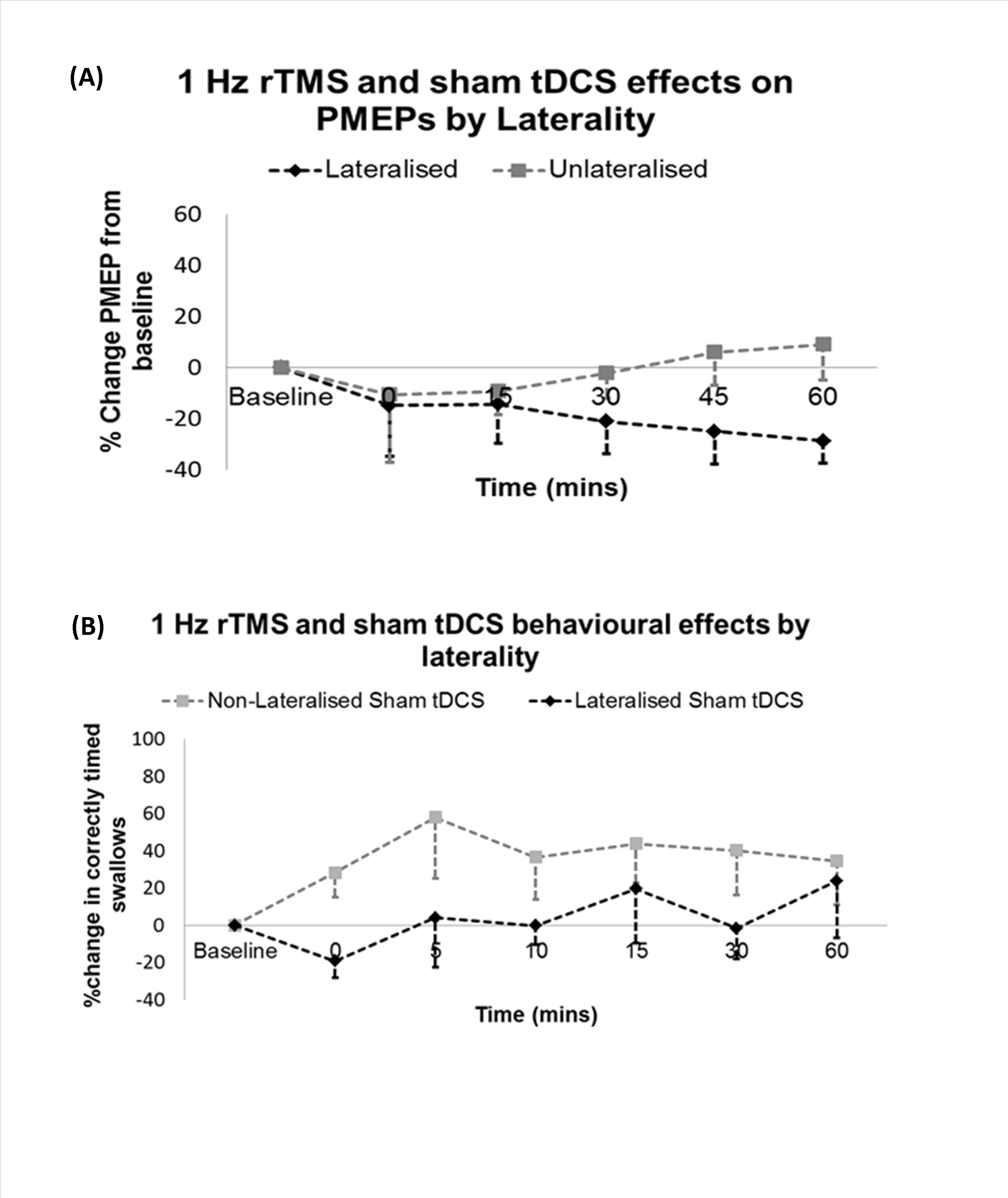

Supplement: Supplementary file 2 [file tjp0592-0695-sd2.tif]
